# Comparative study on epidemiological and etiological characteristics of patients with acute diarrhea with febrile or non-febrile symptoms in China

**DOI:** 10.1186/s40249-023-01108-w

**Published:** 2023-07-04

**Authors:** Tao Wang, Gang Wang, Chun-Xi Shan, Yan-Qun Sun, Xiang Ren, Lin-Jie Yu, Yi-Fei Wang, Sheng-Hong Lin, Xiao-Ai Zhang, Hao Li, Cui-Hong Zhang, Meng-Jie Geng, Wei-Zhong Yang, Li-Ping Wang, Wei Liu, Li-Qun Fang

**Affiliations:** 1 Department of Epidemiology and Biostatistics, School of Public Health, Wuhan University, Wuhan, Hubei People’s Republic of China; 2 State Key Laboratory of Pathogen and Biosecurity, Beijing Institute of Microbiology and Epidemiology, 20 Dong-Da Street, Fengtai District, Beijing, 100071 People’s Republic of China; 3 Nanjing Center for Disease Control and Prevention, Nanjing, People’s Republic of China; 4 Division of Infectious Disease, Key Laboratory of Surveillance and Early-Warning On Infectious Disease, Chinese Center for Disease Control and Prevention, No. 155 Changbai Road, Changping District, Beijing, 102206 People’s Republic of China; 5 Chinese Center for Disease Control and Prevention, Beijing, People’s Republic of China

**Keywords:** Acute diarrhea, Epidemiology, Enteropathogens, Fever, China

## Abstract

**Background:**

Acute diarrhea with fever can potentially represent a more severe form of the disease compared to non-febrile diarrhea. This study was to investigate the epidemiological characteristics and enteric pathogen composition of febrile-diarrheal patients, and to explore factors including pathogens associated with fever by age group.

**Methods:**

A nationwide surveillance study of acute diarrheal patients of all ages was conducted in 217 sentinel hospitals from 31 provinces (autonomous regions or municipalities) in China between 2011 and 2020. Seventeen diarrhea-related pathogens, including seven viruses and ten bacteria, were investigated and their association with occurrence of fever symptoms was assessed using multivariate logistic analysis.

**Results:**

A total of 146,296 patients with acute diarrhea (18.6% with fever) were tested. Th diarrheal children below 5 years had the highest frequency of fever (24.2%), and related to significantly higher prevalence of viral enteropathogens (40.2%) as compared with other age groups (*P* < 0.001). Within each age group, the febrile-diarrheal patients were associated with a significantly higher prevalence of bacterial pathogens than afebrile-diarrheal patients (all *P* < 0.01). There was discrepancy when each pathogen was compared, i.e., nontyphoidal *Salmonella* (NTS) was overrepresented in febrile vs non-febrile patients of all age groups, while the febrile vs non-febrile difference for diarrheagenic *Escherichia coli* (DEC) was only significant for adult groups. The multivariate analysis revealed significant association between fever and infection with rotavirus A among children [odds ratio (*OR*) = 1.60], for DEC in adult groups (*OR* = 1.64), for NTS in both children (*OR* = 2.95) and adults (*OR* = 3.59).

**Conclusions:**

There are significant discrepancy of the infected enteric pathogens in patients with acute diarrhea with fever between age groups, and it is valuable for priority detection of NTS and rotavirus A in patients with children < 5 years old and NTS and DEC in adult patients. The results may be useful in identifying dominant pathogen candidates for the application of diagnostic assays and prevention control.

**Graphical Abstract:**

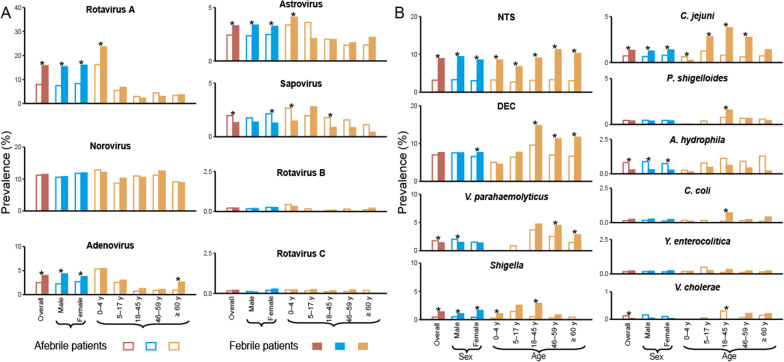

**Supplementary Information:**

The online version contains supplementary material available at 10.1186/s40249-023-01108-w.

## Background

Acute diarrhea is a health problem with high morbidity and mortality rates, causing severe disease burden worldwide [1–3]. According to the estimation from the Global Burden of Diseases, Injuries, and Risk Factors Study (GBD), acute diarrhea was the ninth leading cause of life loss, causing nearly 6.6 billion episodes and 1.5 million deaths in 2019 [4]. Although with an estimated 20.8% decrease in the number of diarrhea-related deaths in the recent decade, diarrhea remains the major contributor to morbidity and mortality in low-income and middle-income countries (LMICs) [5, 6]. Especially in children younger than 5 years, diarrheal disease is the second leading cause of death responsible for 957.5 million diarrheal cases and 498,889 deaths every year [6].

Diarrhea is often accompanied by other symptoms including fever, nausea, vomiting, abdominal pain, fecal urgency, and tenesmus. Among them, fever is one of the major indicators for recognizing severe diarrhea, due to the likely association with complicated illness or invasion of bacterial pathogens into blood, which demands intensive medical management [7, 8]. The empirical treatment of febrile patients tends to be more aggressive than the afebrile one, for example, antibiotic was more often prescribed for febrile patients with diarrhea, for fear of bacterial infection or concurrent viral-bacterial infection [9, 10]. Up to 70% of diarrhea-febrile children were given antibiotics according to a multi-country study [11]. However, indiscriminate prescribing of antibiotics to patients with no bacterial infection could potentially increase antimicrobial resistance and morbidity. There is thus an unmet need to infer differential panel of enteropathogens between febrile and non-febrile diarrheal patients, which may provide an initial qualitative diagnosis to enhance microbiologic diagnosis workflow and thus provide rapid results in clinical practice.

No previous study had ever made such comparison, since rare exhaustive tests of the diarrhea-related pathogens had been performed, as is hindered by the limited geographical and age range of the recruits [12–15]. By analyzing national-based surveillance data on all-age patients with acute diarrhea, we attempt to investigate the enteric pathogen composition among febrile patients with acute diarrhea, to decipher the epidemiological differences from those without fever, and to further explore factors associated with fever.

## Methods

### Surveillance data and processing

Starting from 2011, a nationwide active surveillance was implemented by the Chinese Center for Disease Control and Prevention (China CDC), on the acute diarrheal patients in 31 provinces (autonomous regions or municipalities) (Supplementary methods) [16]. A case of acute diarrhea was defined as the presence of ≥ 3 passages of watery, loose, mucous, or bloody stools within a 24 h period. Both inpatients and outpatients were recruited in the current study. For all participating patients, stool specimens were collected immediately after admission to the hospital and before administration of the therapy (Supplementary methods). A total of 17 commonly seen enteric pathogens, including seven viral pathogens and ten bacterial pathogens, were tested as previously described (Additional file 2: Table S1) [16, 17]. Briefly, rotavirus A antigen was tested by enzyme-linked immunosorbent assay, and reverse transcription-polymerase chain reaction (RT-PCR) was used for G and P genotyping. Other six viruses, including norovirus, adenovirus, astrovirus, sapovirus, rotavirus B and rotavirus C, were tested by polymerase chain reaction (PCR) or RT-PCR. Ten bacterial pathogens were tested by performing isolation in the first step. For *Yersinia enterocolitica*, diarrheagenic *Escherichia coli* (DEC), *Campylobacter jejuni* and *Campylobacter coli*, the isolation was subsequently tested by PCR, and for nontyphoidal *Salmonella* (NTS), *Vibrio parahaemolyticus*, *Vibrio cholerae*, *Aeromonas hydrophila*, *Plesiomonas shigelloides* and *Shigella*, the isolation was subsequently tested by biochemical and serological assays (Additional file 2: Figure S1).

The National Health Commission of the People’s Republic of China determined that the current study was part of public health surveillance and was implemented in accordance with the national surveillance guidelines. The parents/guardians of participants in this study were required to provide verbal consent during enrollment, which was recorded by their physician in each questionnaire. This project and the procedure for obtaining consent were approved by the ethical review committee of China CDC (2015-025).

### Data collection

For each recruited patient, information was obtained that comprised of (1) demography (age, sex, urban or rural residence), (2) clinical symptoms and signs (axillary temperature, stool characteristics, frequency of diarrhea, presence of vomiting, dehydration, respiratory or neurologic symptoms), (3) laboratory testing results, (4) medication during the disease and outcomes. All information was collected through a standardized case reporting form and entered into a standardized database by trained clinicians. All data was uploaded to an online management system structured by the China CDC, sorted to remove redundant data, and checked for incomplete records. The patients were defined as febrile-diarrhea when the axillary temperature measured during the first 24 h after hospital visit was higher than 37.2 °C. Non-febrile patients with acute diarrhea were defined as those who reported no fever before or after a hospital visit for this episode of diarrhea. The sixteen provinces (autonomous regions or municipalities) in southern China and fifteen in northern China were defined according to latitude [18].

### Statistical analysis

For descriptive statistics, frequencies and rates were calculated for categorical variables, while median and interquartile range (IQR) were calculated for continuous variables with abnormal distribution. For the inter-group comparison between patients with and without fever, Chi-squared test or Fisher’s extract test was applied to categorical variables, and Wilcoxon-rank sum test was applied to continuous variables with abnormal distribution. Only patients who had all 17 candidate pathogens tested were used for the pathogen spectrum and clinical symptom analyses (Additional file 2: Figure S2). The prevalence of each tested pathogen was calculated as the number of positive specimens divided by the total number of tests for that pathogen. To compare the proportion of each specific pathogen between febrile and afebrile patients, we calculated the ratio of prevalence (RP) by dividing the prevalence of febrile patients by that of afebrile patients. Binary logistic regression was used to estimate the factors that were significantly associated with the presence of fever. Univariate logistic regression analysis was conducted at the first step, from which all variables with *P* < 0.1 were included into the multivariate analysis. The variance inflation factor (VIF) was calculated to identify the collinearity among the variables. A backward selection procedure was performed to eliminate variables and fit statistics [Akaike information criterion (AIC) and Bayesian Information Criterion (BIC)] to determine the optimal model, which was reached by retaining the variables with *P* < 0.05. Statistical analysis was performed using R statistical software 3.5.3 (Lucent Technologies, Jasmine Mountain, USA), and *P* < 0.05 was statistically significant.

## Results

### Study adherence and demographic characteristics

From January 2011 to December 2020, 149,974 patients with acute diarrhea were recruited, from which 3678 patients were excluded due to inaccurate or missing data. Among the remaining 146,296 patients, 27,160 (18.6%) were accompanied by fever when they were entered into the hospitals (Additional file 2: Figure S2). The demographic and epidemiological characteristics of the diarrheal patients with fever are shown in Table 1. The frequency of fever was slightly higher among male than female patients (19.3% vs 17.5%), decreased with age from 24.2% in the 0–4 age group, 13.0% in the 18–45 group, to 9.9% in the ≥ 60 group, with a linear trend (Cochran-Armitage trend test, *P* = 0.027). Fever was reported with higher proportion in northern region (22.8%) than in southern region (16.5%); in rural (25.7%) than in urban area (16.8%); in outpatients (19.1%) than in inpatients (9.9%); in summer (19.1%), autumn (18.3%) and winter (19.9%) than in spring (16.7%); also relate to a longer delay from disease onset to hospital admission (all *P* < 0.001).

**Table 1:** The demographic and epidemiological characteristics of diarrheal patients with fever in China, 2011–2020

	All diarrheal patients (*n* = 146,296)	Febrile patients *n* (%)^a^ (*n* = 27,160)	*OR*^b^	*P* value^c^
Sex, *n* (%)				< 0.001
Male	85,129	16,466 (19.3, 19.1–19.6)	Reference	
Female	61,167	10,694 (17.5, 17.2–17.8)	0.88	
Age, years, median (IQR)	6 (0.99, 39)	2 (0.93, 24)		< 0.001
0–4, *n* (%)	71,120	17,233 (24.2, 23.9–24.6)	Reference	
5–17, *n* (%)	9454	2171 (23.0, 22.1–23.8)	0.93	
18–45, *n* (%)	34,997	4533 (13.0, 12.6–13.3)	0.47	
46–59, *n* (%)	14,164	1592 (11.2, 10.7–11.8)	0.40	
≥ 60, *n* (%)	16,561	1631 (9.9, 9.4–10.3)	0.34	
Regions, *n* (%)				< 0.001
Northern	48,289	11,018 (22.8, 22.4–23.2)	Reference	
Southern	98,007	16,142 (16.5, 16.3–16.8)	0.67	
Delay from disease onset to hospital admission, days, median (IQR)	2 (2, 4)	3 (2, 4)		< 0.001
Residence, *n* (%)				< 0.001
Urban	111,845	18,832 (16.8, 16.6–17.1)	Reference	
Rural	27,260	7015 (25.7, 25.2–26.3)	1.71	
Case type, *n* (%)				< 0.001
Outpatients	138,250	26,365 (19.1, 18.9–19.3)	Reference	
Inpatients	8046	795 (9.9, 9.2–10.5)	0.47	
Season, *n* (%)				< 0.001
Spring	25,361	4232 (16.7, 16.2–17.2)	Reference	
Summer	43,405	8271 (19.1, 18.7–19.4)	1.18	
Autumn	46,213	8442 (18.3, 17.9–18.6)	1.11	
Winter	31,317	6215 (19.9, 19.4–20.3)	1.24	

^a^The proportion and its 95% confidence interval of febrile-diarrheal patients among all diarrheal patients were shown in parenthesis

^b^Odds ratio (*OR*) values for fever was calculated in two groups

^c^Chi-square test or Fisher’s exact test for comparisons for categorical variables among patients with and without fever, and Wilcoxon rank sum test for continuous variables

In a general manner, the presence of fever was accompanied by more severe disease, featured by higher episodes of diarrhea per day, more common occurrence of watery, mushy, mucous, and bloody stool, as well as more commonly reported vomiting, dehydration, respiratory and neurologic symptoms (all *P* < 0.05) (Table 2). Among them, the most pronounced difference between febrile vs afebrile patients was observed for dehydration (8.4% vs 1.7%) and respiratory symptoms (11.7% vs 4.7%). The age-specific analysis revealed significantly higher frequency of vomiting, dehydration, respiratory and neurologic symptoms in febrile patients within all age groups (all *P* < 0.01), while additional differences in the stool characteristics were mainly observed for the 0–4 years old (*P* < 0.01).

**Table 2:** Demographic and clinical features compared between acute diarrheal patients with fever vs without fever by age groups

	Overall cases (*n* = 40,122)		0–4 years (*n* = 15,705)		5–17 years (*n* = 2322)		18–45 years (*n* = 11,216)		46–59 years (*n* = 4959)		≥ 60 years (*n* = 5920)	
	Febrile	Afebrile	Febrile	Afebrile	Febrile	Afebrile	Febrile	Afebrile	Febrile	Afebrile	Febrile	Afebrile
Sex (No. of male, %)^a^	4113 (59.4)	18,212 (54.9)**	2572 (60.9)	6995 (60.9)	273 (63.2)	1190 (63.0)	771 (59.6)	5289 (53.3)**	239 (50.6)	2103 (46.9)	258 (51.9)	2635 (48.6)
Delay, days, median (IQR)^b^	2 (2–4)	2 (2–3)**	3 (2–4)	3 (2–4)	2 (2–3)	2 (1–3)**	2 (1–3)	2 (1–3)**	2 (1–3)	2 (1–3)	2 (1–3)	2 (1–3)
Frequency of diarrhea, times, median (IQR)^b^	5 (4–7)	5 (3–6)**	5 (4–7)	4 (3–6)**	5 (3–6)	4 (3–6)**	5 (4–8)	5 (4–7)**	6 (4–8)	5 (4–7)	5 (4–8)	5 (4–7)**
Diarrhea > 5 times per day^a^	2921 (42.20)	11,553 (34.85)**	1707 (40.38)	3382 (29.52)**	143 (33.10)	507 (26.91)	599 (46.29)	3808 (38.43)**	242 (51.27)	1779 (39.67)	230 (46.28)	2077 (38.34)*
Stool character												
Watery stool^a^	4838 (69.9)	22,400 (67.5)**	3121 (73.8)	7378 (64.3)**	261 (60.4)	1133 (60.0)	821 (63.5)	7001 (70.6)**	317 (67.1)	3158 (70.4)	318 (64.0)	3730 (68.8)*
Mushy stool^a^	171 (2.5)	502 (1.5)**	136 (3.2)	261 (2.3)**	14 (3.2)	35 (1.9)	12 (0.9)	100 (1.0)	2 (0.4)	45 (1.0)	7 (1.4)	61 (1.1)
Mucous stool^a^	1001 (14.5)	4081 (12.3)**	654 (15.5)	1505 (13.1)**	90 (20.8)	329 (17.4)	132 (10.2)	1064 (10.7)	63 (13.4)	534 (11.9)	62 (12.5)	649 (12.0)
Bloody stool^a^	281 (4.1)	742 (2.2)**	178 (4.2)	230 (2.0)**	30 (6.9)	56 (3.0)**	34 (2.6)	170 (1.7)*	18 (3.8)	110 (2.5)	21 (4.2)	176 (3.3)
Vomiting^a^	2238 (32.3)	6295 (19.0)**	1518 (35.9)	2600 (22.7)**	142 (32.9)	407 (21.6)**	315 (24.3)	1815 (18.3)**	129 (27.3)	731 (16.3)**	134 (27.0)	742 (13.7)**
Respiratory symptoms^a^	807 (11.7)	1549 (4.7)**	747 (17.7)	1397 (12.2)**	33 (7.6)	52 (2.8)**	11 (0.9)	24 (0.2)**	7 (1.5)	17 (0.4)**	9 (1.8)	59 (1.1)
Dehydration^a^	585 (8.5)	578 (1.7)**	421 (10.0)	223 (1.9)**	26 (6.0)	30 (1.6)**	68 (5.3)	159 (1.6)**	32 (6.8)	80 (1.8)**	38 (7.7)	86 (1.6)**
Neurologic symptoms^a^	113 (1.6)	212 (0.6)**	61 (1.4)	96 (0.8)**	6 (1.4)	6 (0.3)*	2 (2.0)	46 (0.5)**	9 (1.9)	29 (0.7)**	11 (2.2)	35 (0.7)**

^a^Chi square test or Fisher’s extract test for comparisons of differences between fever and afebrile cases

^b^Wilcoxon test for comparisons of differences between febrile and afebrile cases

All statistical tests were two-sided and significant differences were showed by asterisks (*P < 0.05; **P < 0.01). *IQR* Interquartile range

### Prevalence of virus pathogens in febrile vs afebrile patients

Altogether 40,122 diarrheal patients (6922 febrile and 33,200 afebrile) were recruited from 154 hospitals, who had all 17 pathogens tested (Additional file 2: Figure S2). In total, 31.3% (2163/6922) of the febrile-diarrheal patients had at least one virus positive detection, with the highest frequency determined in children < 5 years old (40.2%, 1699/4227), followed by 22.2% (96/432) in 5–17 years adolescents, 18.0% (85/472) in 46–59 years adults, 15.9% (79/497) in the ≥ 60 years elderly people, and 15.8% (204/1294) in 18–45 years adults. Significantly higher overall prevalence of pathogens was seen in febrile than afebrile-diarrheal in all patients (31.3%, 2163/6922 vs 23.7%, 7875/33,200; *P* < 0.001), also within 0–4 years group (40.2%, 1699/4227 vs 35.7%, 4094/11,478;* P* < 0.001), whereas not for the other age groups (Additional file 2: Table S2).

**Fig. 1:**
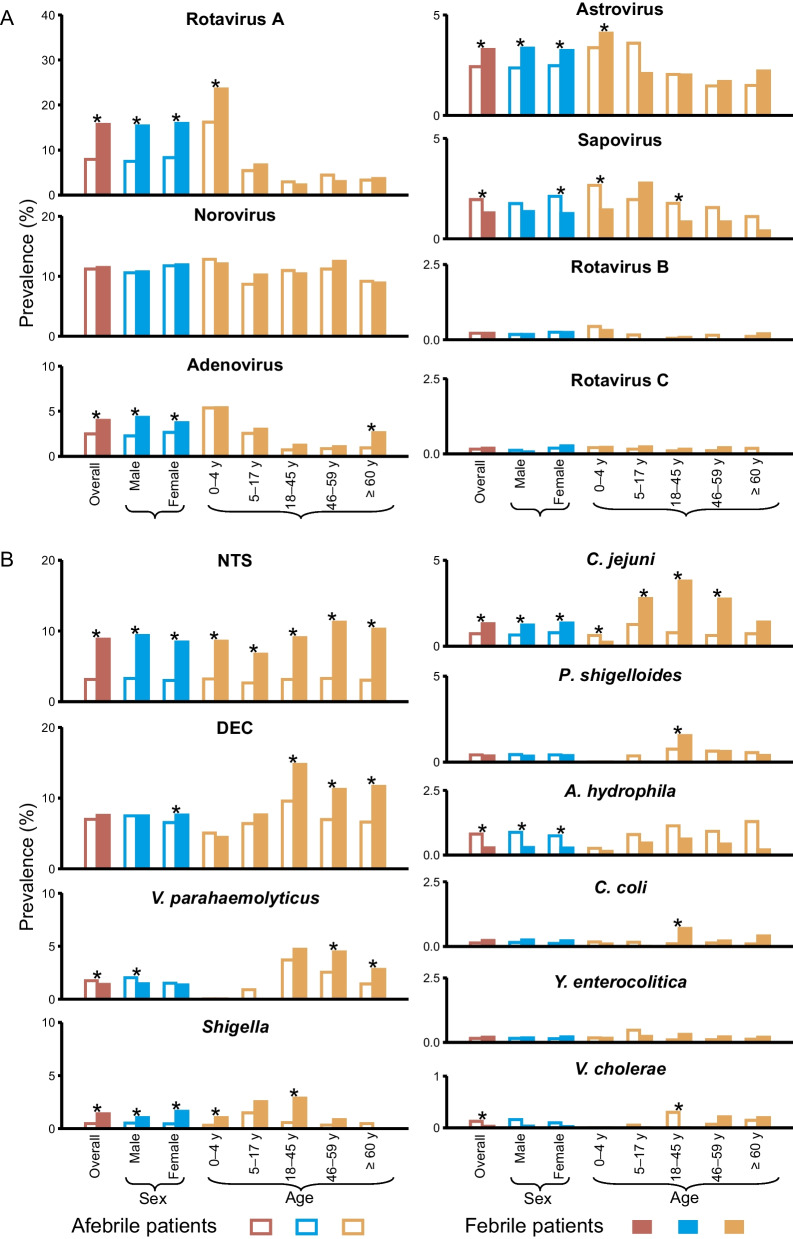
Prevalence of 17 common enteropathogens between diarrheal patients with and without fever. **A** Viral pathogens. **B** Bacterial pathogens. The length of the bar indicates the prevalence of each pathogen. The unfilled and solid bars indicate the afebrile and febrile patients, respectively. Sex differences are marked by blue bars and age differences by yellow bars. *Significant difference with *P* < 0.05 compared between febrile and afebrile patients by performing Chi-square test or Fisher’s exact test. The prevalence of each pathogen is supplemented in Additional file 2: Table S2. *DEC* diarrheagenic *Escherichia coli*; *NTS* nontyphoidal *Salmonella*; y Years

Among all the recruited diarrheal patients, norovirus was most determined, followed by rotavirus A > adenovirus > astrovirus > sapovirus. Compared with afebrile-diarrheal patients, febrile patients had a significantly higher prevalence of rotavirus A (15.7% vs 7.9%), adenovirus (4.0% vs 2.5%) and astrovirus (3.3% vs 2.4%), while comparable prevalence of norovirus and significantly lower prevalence of sapovirus (1.3% vs 2.0%). When disaggregated by age groups, the febrile-afebrile difference remained significant for rotavirus A, astrovirus, and sapovirus only among pediatric patients, and the difference for adenovirus remained only among the ≥ 60 years (all *P* < 0.05). Most of these differences were likewise observed for the male and female gender, except for sapovirus, for which no febrile/afebrile difference was observed for females (Fig. 1, Additional file 2: Table S2).

### Prevalence of bacterial pathogens in febrile vs afebrile patients

In total, 20.2% (1401/6922) of the febrile-diarrheal patients had at least one bacterial positive detection, with the highest prevalence determined in 18–45 years adults (33.9%, 439/1294), followed by 30.1% (142/472) in 46–59 years adults, 26.2% (130/497) in ≥ 60 years elderly people, 19.2% (83/432) in 5–17 years adolescents, and 14.4% (607/4227) in the children < 5 years old (Additional file 2: Table S2). Significantly higher rate was seen in febrile than afebrile patients as a whole (20.2% vs 13.9%, *P* < 0.001) and this difference remained significant for all the five age groups (Additional file 2: Table S2, all *P* < 0.01), with the febrile/afebrile RP increased as patients get old (Additional file 2: Table S3). The highest bacterial prevalence was observed for NTS, followed by DEC > *V. parahaemolyticus* > *Shigella* > *C. jejuni* > *P. shigelloides*. Compared with afebrile-diarrheal patients, febrile patients had significantly higher prevalence of NTS (8.8% vs 3.1%), DEC (7.6% vs 7.0%), *Shigella* (1.4% vs 0.5%) and *C. jejuni* (1.3% vs 0.7%), and significantly lower rate of *V. parahaemolyticus* (1.4% vs 1.8%), *A. hydrophila* (0.3% vs 0.8%) and *V. cholerae* (0.03% vs 0.1%) (Fig. 1, Additional file 2: Table S2). When disaggregated by age group, the febrile-afebrile difference remained significant for NTS in all age groups and for DEC and *C. jejuni* in all three adult groups. It’s notable that for both NTS and DEC, the extent of difference increased with age, for example, the febrile/afebrile RP of DEC was 0.89 in 0–4 age group, increased to 1.19 in 5–17 group, 1.54 in 18–45 group, 1.62 in 46–60 group and 1.77 in the ≥ 60 group. For the other enteric bacteria, however, the difference between fever and non-fever patients was significant only within a small number of groups (all *P* < 0.05). Most of the febrile/afebrile differences were likewise observed for male and female gender, except for DEC, no febrile/afebrile difference was observed for females (Fig. 1, Additional file 2: Table S2-3).

### Co-infections in febrile vs afebrile patients

**Fig. 2:**
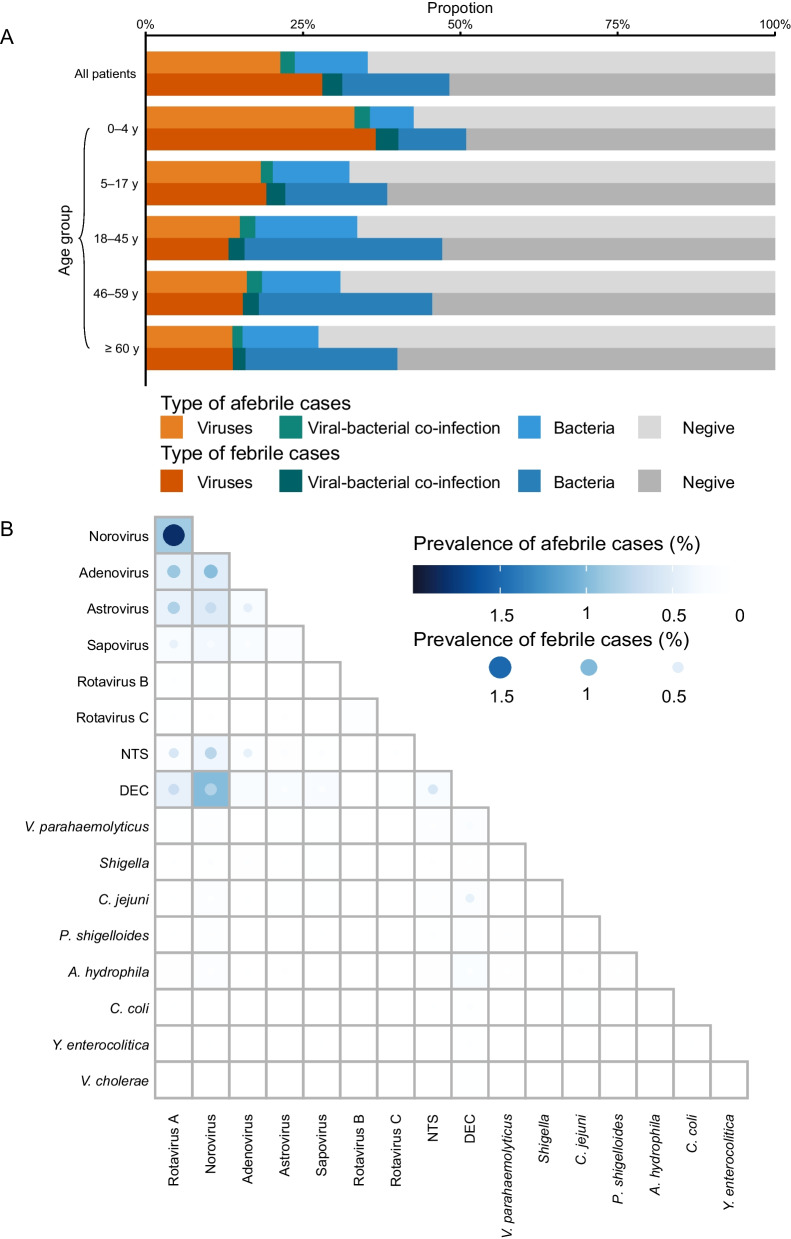
Viral-bacterial coinfection in diarrheal patients with and without fever, in China, 2011‒2020. **A** Proportion of viruses, bacteria, and viral-bacteria coinfections among febrile and afebrile patients with diarrhea, respectively. **B** Coinfection pattern of viruses and bacteria for febrile and afebrile patients. The prevalence of coinfection is supplemented in Additional file 2: Tables S2 and S3. *DEC* diarrheagenic *Escherichia coli*; *NTS* nontyphoidal *Salmonella*

Co-infection with ≥ 2 viruses was seen in 4.2% of the febrile patients, significantly higher than that of the afebrile patients (2.4%, *P* < 0.001, Additional file 2: Table S2). This difference was more pronounced among elderly people (with febrile/afebrile RP of 2.14, Additional file 2: Table S3) than the other age groups. Viral co-infection primarily occurred for rotavirus A-norovirus, rotavirus A-adenovirus and adenovirus-norovirus, with rotavirus A-norovirus, rotavirus A-adenovirus, rotavirus A-astrovirus, rotavirus A-sapovirus, norovirus-adenovirus, norovirus-astrovirus and adenovirus-astrovirus viral pair significantly higher among febrile than the afebrile patients (Fig. 2B, Additional file 2: Table S4).

Coinfection with ≥ 2 bacteria was seen in 1.3% of the febrile patients, significantly higher than that of the afebrile patients (0.8%, *P* < 0.001, Additional file 2: Table S2). This difference was more pronounced among 46–59 years adult group (with febrile/afebrile RP of 3.07, Additional file 2: Table S3) than the other age groups. Bacterial coinfection primarily occurred for DEC-NTS, DEC-*C. jejuni*, with DEC-NTS, DEC-*C. jejuni* and DEC- *Shigella* significantly higher among febrile than afebrile patients (Fig. 2B, Additional file 2: Table S4, all *P* < 0.01).

The viral-bacterial coinfection rate in the febrile patients was 3.2%, significantly higher than that of the afebrile groups (2.3%, *P* < 0.001, Additional file 2: Table S2). This febrile/afebrile difference increased as the age increased, with the highest RP observed among elderly people (1.71), vs 1.30, 1.48, 1.60, and 1.41 in the other four age groups, respectively (Fig. 2A, Additional file 2: Table S3). Viral-bacterial coinfection primarily occurred among DEC, NTS and rotavirus A, norovirus, with DEC-rotavirus A, NTS-rotavirus A, NTS-norovirus, NTS-adenovirus, NTS-astrovirus, NTS-rotavirus C, and *Shigella*-norovirus significantly more frequent in febrile patients than in afebrile patients, by contrast, with DEC-norovirus lower in febrile patients (Fig. 2B, Additional file 2: Table S4).

### Factors associated with fever in febrile patients

**Fig. 3:**
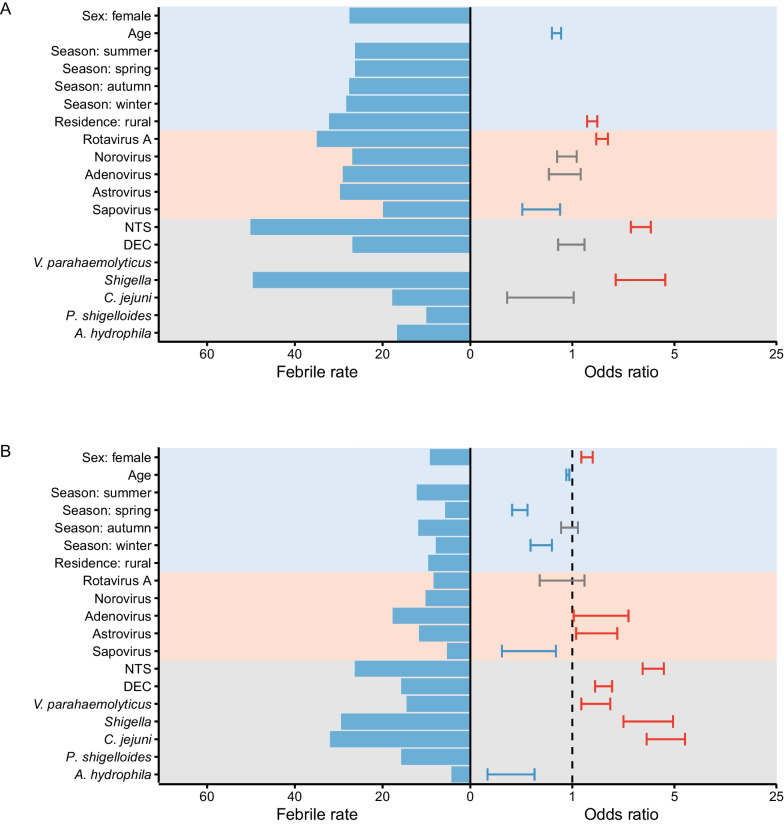
Factors associated with fever in diarrheal disease among children (**A**) and adults (**B**). The multivariable logistic regression was performed to include demographic characteristics (sex, age, residential regions and season of disease), and results of pathogen detection (only 12 enteropathogens with positive detection in more than 100 cases were included). The bars indicate the rate of febrile among patients in each subgroup; The point and error bar show the adjusted odds ratios (*OR*s) and 95% confidence interval (*CI*) associated with fever, with red color indicating the effect with *OR* > 1; blue color indicating effect with *OR* < 1; grey indicating effect with no significance. *DEC* diarrheagenic *Escherichia coli*; *NTS* nontyphoidal *Salmonella*

Altogether 16 variables were entered into the logistic regression model, which included demographic characteristics (sex, age, residential region and season of disease), and test results of 12 enteropathogens (the positive detection of each pathogen was observed in ≥ 100 cases). The logistic model was conducted in children < 18 years and adults, respectively, given their differential host response to enteropathogens. For the children group, lower age and residence in rural areas were related to increased risk of fever [adjusted odds ratio (a*OR*): 1.19, 95% confidence interval (*CI*): 1.12–1.25; a*OR*: 1.36, 95% *CI*: 1.26–1.48]. For the adult group, male gender, lower age, and infection in summer, autumn and winter seasons were significantly associated with increased risks of fever (a*OR*: 1.26, 95% *CI*: 1.15–1.38; a*OR*: 1.04, 95% *CI*: 1.03–1.06; a*OR*: 2.07, 95% *CI*: 1.77–2.43; a*OR*: 2.04, 95% *CI*: 1.75–2.40; a*OR*: 1.43, 95% *CI*: 1.18–1.72) (Fig. 3, Additional file 2: Table S5).

When pathogen detection was individually considered, increased incidence of fever was significantly associated with positive detection of rotavirus A in children (a*OR*: 1.60, 95% *CI*: 1.46–1.75), while sapovirus was significantly associated with decreased risk of fever in both children and adults (a*OR*: 0.67, 95% *CI*: 0.51–0.88; a*OR*: 0.53, 95% *CI*: 0.31–0.84). Infection with NTS and *Shigella* was associated with an increased incidence of fever in both children and adults, with a higher effect observed for NTS. The association between fever and DEC, *V. parahaemolyticus* and *C. jejuni*, was observed only among the adults, consistent with the results from the inter-group comparison (Fig. 3; Additional file 2: Table S5).

## Discussion

These comparisons of symptoms between febrile and afebrile patients suggest that although not necessarily accompanied by diarrhea, fever represents a severe form of the disease and may indicate a systemic illness with clinical symptoms extending beyond the digestive tract, and should be closely monitored to prevent adverse outcomes (Table 2). The current study represents, to the best of our knowledge, the first of its kind to use long-term surveillance data to interpret the enteropathogen profile of diarrheal patients with fever. It’s not surprising to observe a high prevalence of enteric pathogens in the febrile patients, whether viruses or bacteria, with a single infection or coinfection. Age-specific differences, however, are a notable finding. Compared with afebrile patients, higher prevalence of viral pathogens among febrile patients were only seen among < 5 years children, indicating a more remarkable role of gastroenteric virus (primarily rotavirus A) in fever disease in children, while not for other age groups (Additional file 2: Table S2). While higher bacterial prevalence among febrile patients was seen across all age groups, we observed more prominent differences among adults, mainly with two bacteria (DEC and NTS) responsible for the differences. All of these associations with fever have been verified by multivariate analysis, and thus our study represents credible evidence for a pathogenic diagnosis in diarrhea.

Notably, we disclosed a significant association between increased incidence of fever for the < 5 years old patients if they were infected with rotavirus. As has been well accepted, rotavirus A plays a predominant role in pediatric diarrhea, which can cause a wide range of diseases, ranging from watery diarrhea to systematic infection that can even result in death [19]. The fact that rotavirus A is involved in severe cases, but that co-infection with other enteric pathogens also appears to aggravate the severity of diarrhea in children has been established following investigations in southwest China [20]. In older children and adults, by contrast, rotavirus A infection causes limited illness. This was also consistent with one prospective study showing association between positive detection of rotavirus RNA and rotavirus antigen in both serum and stools and increased incidence of fever and more severe vomiting, both indicative of a systematic infection [7]. In addition, sapovirus was significantly associated with a decreased risk of fever in both children and adults. This is consistent with previous studies of children infected with sapovirus, which have shown that the incidence of acute gastroenteritis fever caused by sapovirus infection is lower than that caused by other pathogenic infections [21, 22].

There has been a traditional view that fever is suggestive of bacterial diarrhea [23], which can be further refined by the current finding. The association between each enteric pathogen and fever varies across age groups and may be useful in identifying dominant pathogen candidates and priority targets for applying differential diagnosis, prevention and control. For example, while all age groups with bacterial infections were associated with a higher odd of having a higher febrile disease, the effect was more pronounced in adults. Among the commonly seen bacterial enteropathogens, NTS and DEC appear as the dominant ones in patients with fever, which can assist in informed diagnostic algorithms in clinical management. This finding is in agreement with a previous study in sub-Saharan Africa, where a high prevalence of invasive NTS in febrile patients was associated with a high case fatality rate of 20.6% among all age groups [24]. In addition, the treatment of diarrheal cases with fever will be compromised by the increasing number of antimicrobial resistances in China [25].

Higher prevalence of viral, bacterial and viral-bacterial coinfection was consistently observed in febrile-diarrheal patients than in afebrile patients (Additional file 2: Table S3). In congruent with previous studies in Europe, diarrheal patients with coinfection had more severe clinical presentation, especially for children [26, 27]. Co-infection pattern that was related to increased risk of fever is also specified in the current study and should be given priority in medical management and more aggressive treatment. In addition, due to the high prevalence of co-infections, urgent development of laboratory methods to assess multi-microbial infections is recommended for better prevention and treatment strategies to control diarrhea [28].

Fever, as a classic response to infection, and manifestation of cytokine release in response to a variety of stimuli, might be beneficial for the host’s response to infection [22]. For example, in children following rotavirus gastroenteritis, significantly increased serum levels of IL-6 and TNF have been reported in fever patients more than in those without [19]. While for NTS, an invasive disease might occur due to lower inflammatory reaction in the intestine, ensued by less activation of the host immune response, which benefits dissemination of NTS beyond the gut and gut-associated lymphoid tissue [29].

We also noticed a significant association between rural area residents and a higher prevalence of fever in the children group. It’s hypothesized that higher exposure to unsafe water, livestock and poultry, poor hygiene conditions in rural areas might render more exposure to a high variety and thus coinfection of enteric pathogens [30, 31]. Delayed access to health care services was also contributory to higher risk of serious clinical outcomes, even infected with the same enteric pathogens [32], this was also reflected by the current association between longer delay and more fever incidence. More occurrence of fever was noticed in summer and autumn for adults, which might be due to the higher prevalence of bacterial infection in these seasons, leading to an indirect association between fever and season [16]. Our previous study verified a summer‒autumn seasonality of bacterial diarrhea, which was mainly caused by DEC, *Shigella*, NTS and *A. hydrophila* in China [16].

The study is subject to several limitations. Although 17 pathogens were tested, there were other missed or unknown pathogens, for example, *Arcobacter* or *Laribacter*, two parasitic pathogens that could have been the cause of the current episode of acute diarrhea, that failed to be exhaustively tested. Another limitation lies in that the causal relationship between enteropathogens and symptoms cannot be determined due to the inherent limitations of the observational study design. Moreover, individual-level medical history information, such as comorbidities, body mass index (BMI), etc., are potential factors that may influence fever incidence and warrant further in-depth data curation and analysis.

## Conclusions

Our study revealed a high prevalence of enteric pathogens among the febrile patients, regardless of viral or bacterial origin. Enteric virus (primarily rotavirus A) was the leading cause of febrile-diarrhea in children under five, while bacterial pathogens were significantly overrepresented in febrile patients across all age groups, especially among adults and mainly due to two bacteria (nontyphoidal *Salmonella* and diarrheagenic *Escherichia coli*). We believe that the current study may provide an initial qualitative diagnosis to enhance microbiologic diagnosis workflow, which may be useful in identifying dominant pathogen candidates and priority targets for the application of diagnostic assays, therapeutic modalities, and preventive control in this severe form of diarrheal disease. When rapid diagnosis results could be offered in clinical practice, the misuse of antibiotics might be largely avoided.

## Supplementary Information

**Additional file 1.** The Chinese Centers for Disease Control and Prevention (CDC) Etiology of Diarrhea Surveillance Study Team.

**Additional file 2.** Supplementary methods.

## Data Availability

Raw data are not publicly available and are protected due to data privacy laws, which were used under license for the current study, but are available upon reasonable request to the corresponding author and with permission from the data provider (Li-Ping Wang). The request will be responded to within 1 week.

## Abbreviations

NTS: Nontyphoidal *Salmonella*
DEC: Diarrheagenic *Escherichia coli*
*OR*: Odds ratio
GBD: Global Burden of Diseases
LMICs: Low-income and middle-income countries
China CDC: Chinese Center for Disease Control and Prevention
RT-PCR: Reverse transcription-polymerase chain reaction
IQR: Interquartile range
RP: Ratio of prevalence
VIF: Variance inflation factor
AIC: Akaike information criterion
BIC: Bayesian information criterion
*CI*: Confidence interval
BMI: Body mass index
