# Supplementary material for: Comparative study on epidemiological and etiological characteristics of patients with acute diarrhea with febrile or non-febrile symptoms in China
Source: Infect Dis Poverty. 2023 Jul 4;12:62. doi: 10.1186/s40249-023-01108-w (PMC10318681; doi:10.1186/s40249-023-01108-w)
Supplement: Supplementary file 1 — Additional file 1. The Chinese Centers for Disease Control and Prevention (CDC) Etiology of Diarrhea Surveillance Study Team. [file 40249_2023_1108_MOESM1_ESM.docx]

**Additional file 1**

**The Chinese Centers for Disease Control and Prevention (CDC) Etiology of Diarrhea Surveillance Study Team**

1. Chinese Center for Disease Control and Prevention, Beijing, China: Wei-Zhong Yang; George F. Gao.

2. Division of Infectious Disease, Key Laboratory of Surveillance and Early warning on Infectious Disease, Chinese Center for Disease Control and Prevention, Beijing, China: Zhong-Jie Li; Li-Ping Wang; Xiang Ren; Yi-Fei Wang; Sheng-Hong Lin; Cui-Hong Zhang; Meng-Jie Geng.

3. National Institute for Communicable Disease Control and Prevention, Chinese Center for Disease Control and Prevention, Beijing, China: Xin Wang; Huai-Qi Jing.

4. National Institute for Viral Disease Control and Prevention, Chinese Center for Disease Control and Prevention, Beijing, China: Wen-Bo Xu; Ai-Li Cui.

5. National Institute of Parasitic Diseases, Chinese Center for Disease Control and Prevention, Shanghai, China: Yu-Juan Shen; Yan-Yan Jiang.

6. Center of Disease Prevention and Control in Pudong New Area of Shanghai, Shanghai, China: Qiao Sun; Li-Peng Hao; Chu-Chu Ye.

7. State Key Laboratory of Pathogen and Biosecurity, Beijing Institute of Microbiology and Epidemiology, Beijing, China: Wei Liu; Xiao-Ai Zhang.

8. The Institute for Disease Prevention and Control of PLA, Beijing, China: Liu-Yu Huang; Yong Wang; Wen-Yi Zhang.

9. Wuhan University, Wuhan, China: Ying-Le Liu; Jian-Guo Wu; Qi Zhang.

10. Tongji Hospital, Tongji Medical College, Huazhong University of Science and Technology, Wuhan, China: Wei-Yong Liu; Zi-Yong Sun.

11. Hubei Provincial Center for Disease Control and Prevention, Wuhan, China: Fa-Xian Zhan.

12. Jiangxi Provincial Center for Disease Control and Prevention, Nanchang, China: Ying Xiong.

13. Gansu Provincial Center for Disease Control and Prevention, Lanzhou, China: Lei Meng; De-Shan Yu.

14. Qinghai Provincial Center for Disease Control and Prevention, Xining, China: Chun-Xiang Wang; Sheng-Cang Zhao.

15. Inner Mongolia Autonomous Region Comprehensive Center for Disease Control and Prevention, Hohhot, China: Wen-Rui Wang; Xia Lei.

16. Lanzhou University, Lanzhou, China: Juan-Sheng Li.

17. Lanzhou Center for Disease Control and Prevention, Lanzhou, China: Yu-Hong Wang; Yan Zhang.

18. Baiyin Center for Disease Control and Prevention, Baiyin, China: Jun-Peng Yang; Yan-Bo Wang.

19. Tianshui Center for Disease Control and Prevention, Tianshui, China: Fu-Cai Quan; Zhi-Jun Xiong.

20. Wuwei Center for Disease Prevention and Control, Wuwei, China: Li-Ping Liang; Quan-E Chang.

21. Qingyang Center for Disease Control and Prevention, Qingyang, China: Yun Wang; Ping Wang.

22. Liaoning Provincial Center for Disease Control and Prevention, Shenyang, China: Zuo-Sen Yang; Ling-Ling Mao.

23. Tianjin Center for Disease Control and Prevention, Tianjin, China: Jia-Meng Li; Li-Kun Lv.

24. Heilongjiang Provincial Center for Disease Control and Prevention, Harbin, China: Jun Xu; Chang Shu.

25. Zhejiang University, Hangzhou, China: Xiao Chen; Yu Chen.

26. Zhejiang Center for Disease Control and Prevention, Hangzhou, China: Yan-Jun Zhang.

27. Jiangsu Provincial Center for Disease Control and Prevention, Nanjing, China: Lun-Biao Cui.

28. Fujian Center for Disease Control and Prevention, Fuzhou, China: Kui-Cheng Zheng.

29. Beilun People’s Hospital, Ningbo, China: Xing-Guo Zhang.

30. Shanghai Municipal Center for Disease Control and Prevention, Shanghai, China: Xi Zhang; Li-Hong Tu.

31. Shanghai Public Health Clinical Center, Shanghai, China: Zhi-Gang Yi.

32. Yunnan Center for Disease Control and Prevention, Kunming, China: Wei Wang; Shi-Wen Zhao; Xiao-Fang Zhou.

33. Sichuan University, Chengdu, China: Xiao-Fang Pei; Tian-Li Zheng.

34. Chongqing Medical University, Chongqing, China: Xiao-Ni Zhong.

35. Chongqing Center for Disease Control and Prevention, Chongqing, China: Qin Li; Hua Ling.

36. Guizhou Center for Disease Control and Prevention, Guiyang, China: Ding-Ming Wang; Shi-Jun Li.

37. Sichuan Province Center for Disease Control and Prevention, Chengdu, China: Shu-Sen He.

38. Sun Yat-sen University, Guangzhou, China: Meng-Feng Li; Jun Li; Xun Zhu.

39. Guangdong Provincial Center for Disease Control and Prevention, Guangzhou, China: Chang-Wen Ke; Hong Xiao.

40. Guangzhou Municipal Center for Disease Control and Prevention, Guangzhou, China: Biao Di; Ying Zhang.

41. Zhujiang Hospital, Southern Medical University, Guangzhou, China: Hong-Wei Zhou; Nan Yu.

42. Jinan University, Guangzhou, China: Hong-Jian Li; Fang Yang.

43. The Third People’s Hospital of Shenzhen, Shenzhen, China: Fu-Xiang Wang; Jun Wang.
