# Supplementary material for: Comparative study on epidemiological and etiological characteristics of patients with acute diarrhea with febrile or non-febrile symptoms in China
Source: Infect Dis Poverty. 2023 Jul 4;12:62. doi: 10.1186/s40249-023-01108-w (PMC10318681; doi:10.1186/s40249-023-01108-w)
Supplement: Supplementary file 2 — Additional file 2. Supplementary methods. [file 40249_2023_1108_MOESM2_ESM.docx]

**Additional file 2**

Supplement to: Tao Wang, et al. Comparative study on epidemiological and etiological characteristics of patients with acute diarrhea with febrile or non-febrile symptoms in China.

**Supplementary methods**

**The active surveillance system**

Between 2011 and 2020, an active surveillance on patients with acute diarrhea was administered in 217 sentinel hospitals and 93 reference laboratories in all 31 provinces (autonomous regions or municipalities) in the Chinese mainland which was managed by Chinese Center for Diseases Control and Prevention (China CDC). The numbers of sentinel hospitals and reference laboratories were determined in proportion to the total population size within each ecological region and the sentinel hospitals were chosen after careful consideration for the capacities of surveillance and laboratory testing and the representativeness of geographical locations. All participating hospitals and laboratories used a surveillance protocol that included guidelines for patient enrollment, specimen collection, laboratory testing, data management and other related standard operating procedures (SOP) that were developed by China CDC. The patients were recruited at the emergency department, infection department, pediatrics and intestinal clinics. Patients with other main complaints than acute diarrhea was excluded from the study. Patients referred from other hospitals or patients not initially diagnosed in sentinel hospitals or patients with non-infectious disease were excluded from this study.

**Specimen collection**

Stool specimens were collected immediately after the participating patients were admitted into the hospital and before therapy was administered. For virological testing, stool was collected in sterilized containers without preservatives and tested as soon as possible, and if not, were stored at -80 °C until tested. For bacteriological testing, stool specimens were collected using five sterilized cotton swabs and immediately plated onto culture medium, and if not, were placed in Cary Blair Medium at 4 °C for transporting to the laboratory. Polymerase Chain Reaction (PCR) was performed to identify the pathotypes of DEC by targeting their unique virulence genes. The target genes included *aaf* Ⅱ for EAEC, *eae* and *bfp* for tEPEC, *eae* or *bfp* for aEPEC, *lt* and/or *st* Ⅱ for ETEC, *virF* and *ipaH* for EIEC, and *eae* and *stx*1 and/or *stx*2 for EHEC. The primer sequences, annealing temperature, and amplicon size were detailed in our previous literature^[1, 2]^.

**Supplementary References**

1. Wang LP, Zhou SX, Wang X, Lu QB, Shi LS, Ren X, et al. Etiological, epidemiological, and clinical features of acute diarrhea in China. Nat Commun. 2021;12(1):2464.

2. Zhou SX, Wang LP, Liu MY, Zhang HY, Lu QB, Shi LS, et al. Characteristics of diarrheagenic Escherichia coli among patients with acute diarrhea in China, 2009‒2018. J Infect. 2021;83(4):424-32.


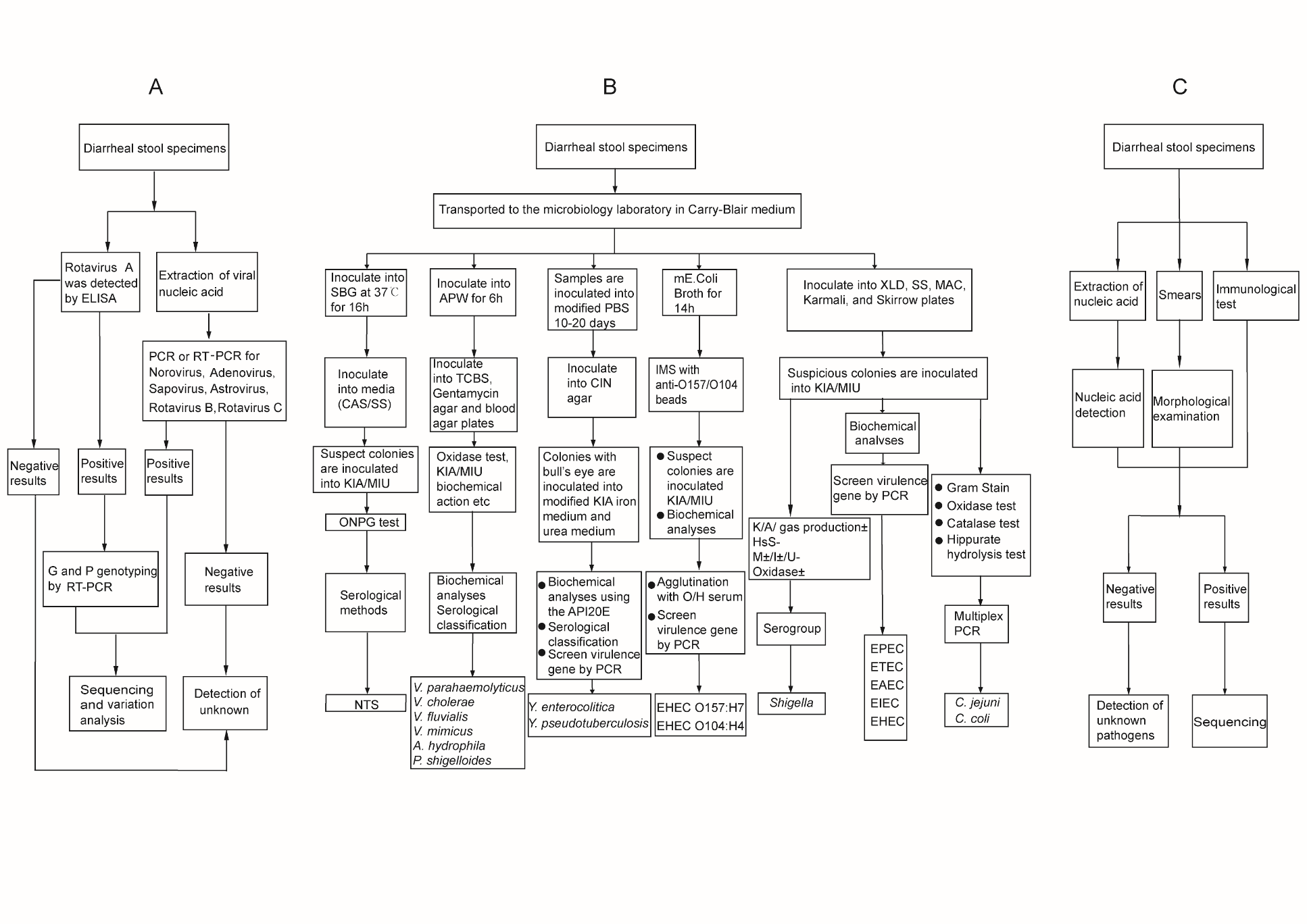


**Figure S1. The flowchart of laboratory tests.** (A) Flow chart for viral pathogens test. (B) Flow chart for bacterial pathogens test. (C) Flow chart for parasitical pathogens test. SBG=selenite brilliant green; APW=alkaline peptone water; PBS=phosphate buffered saline; XLD=xylose lysine desoxycholate; SS=Salmonella-Shigella; MAC=MacConkey; CAS=Chrom agar Salmonella; TCBS=thiosulfate-citrate-bile salts-sucrose; CIN=Cefsulodin-irgasan-novobiocin; KIA=Kligler iron agar; MIU=motility indole urea; ONPG=the ortho-nitrophenol test for beta-galactosidase production^[1]^.


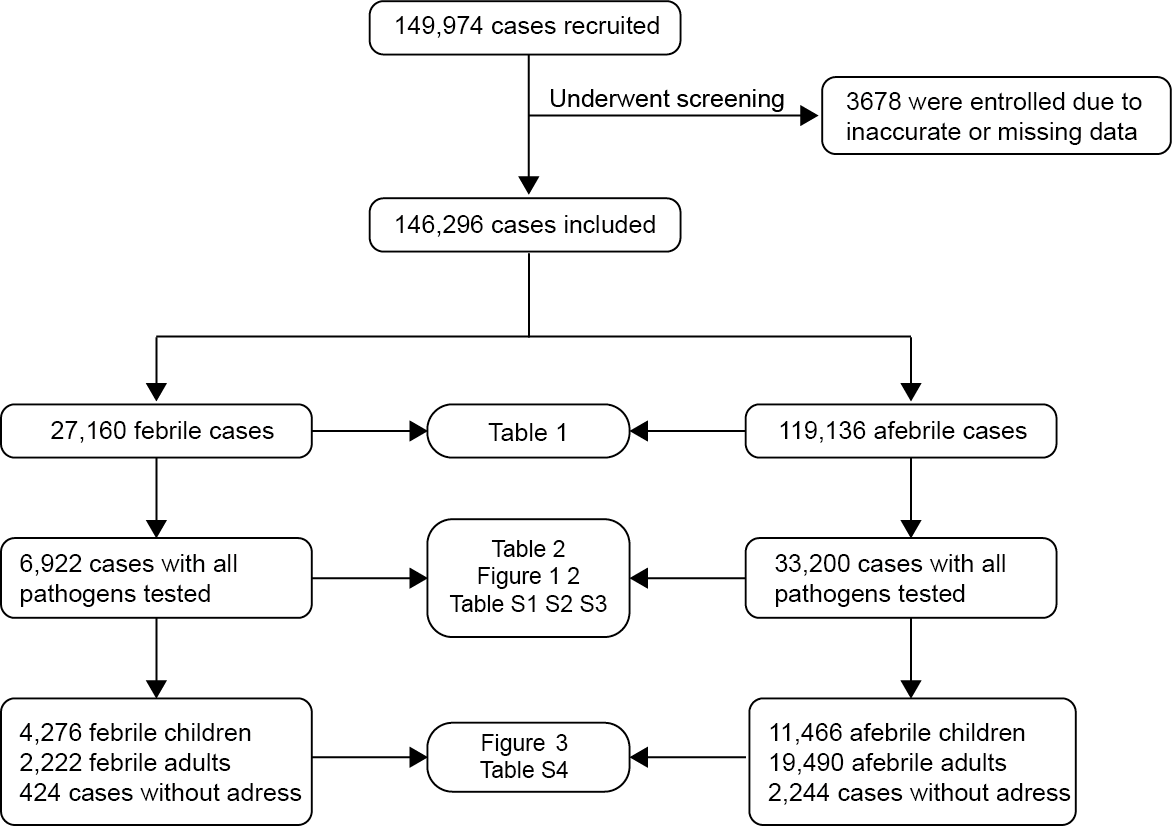


**Figure S2. A flowchart of the data collection and sorting procedures.** This flow diagram summarizes the number of patients with acute diarrhea for each analysis in this study.

**Table S1. Primers and sequence information for PCR used in diarrheal illnesses surveillance of China^[1]^.**

Table S1-1. Primers and sequence information used for characterizing G/P genotypes of Group A Rotavirus by RT-PCR.

|  | **Genotype** | **Primer** | **Sequence (5’-3’)**^c^ | **Product Size (bp)** | **Position** |
| --- | --- | --- | --- | --- | --- |
| G-typing | G | VP7F | ATGTATGGTATTGAATATACCAC | 881 | 51-71 |
|  |  | VP7R | AACTTGCCACCATTTTTTCC |  | 914-932 |
|  |  |  |  |  |  |
|  | G1 | aBT1 | CAAGTACTCAAATCAATGATGG | 618 | 314-335 |
|  | G2 | aCT2 | CAATGATATTAACACATTTTCTGTG | 521 | 411-435 |
|  | G3 | G3 | ACGAACTCAACACGAGAGG | 682 | 250-269 |
|  | G4 | aDT4 | CGTTTCTGGTGAGGAGTTG | 452 | 480-499 |
|  | G8 | aAT8 | GTCACACCATTTGTAAATTCG | 754 | 178-198 |
|  | G9 | G9 | CTTGATGTGACTAYAAATAC | 179 | 757-776 |
| P-typing | P | VP4F | TATGCTCCAGTNAATTGG | 663 | 132-149 |
|  |  | VP4R | ATTGCATTTCTTTCCATAATG |  | 775-795 |
|  |  |  |  |  |  |
|  | P[4] | 2T-1 | CTATTGTTAGAGGTTAGAGTC | 362 | 474-492 |
|  | P[6] | 3T-1 | TGTTGATTAGTTGGATTCAA | 146 | 259-278 |
|  | P[8] | 1T-1D | TCTACTGGRTTRACNTGC | 224 | 339-356 |
|  | P[9] | 4T-1 | TGAGACATGCAATTGGAC | 270 | 385-402 |
|  | P[10] | 5T-1 | ATCATAGTTAGTAGTCGG | 462 | 575-594 |
|  | P[11] | P[11] | GTAAACATCCAGAATGTG | 191 | 305-323 |

Table S1-2. Primers and sequence information used in PCR for viral agents.

| **Organism** | **Primers and sequence information** | | | |
| --- | --- | --- | --- | --- |
|  | **Primer** | **Polarity** | **Sequence (5’-3’)** | **Product Size (bp)** |
| Rotavirus B | B5-2 | + | GGCAATAAAATGGCTTCATTGC | 814 |
|  | B3-3 | - | GGGTTTTTACAGCTTCGGCT |  |
| Rotavirus C | NG8S1 | + | ATTATGCTCAGACTATCGCCAC | 352 |
|  | NG8A2 | - | GTTTCTGTACTAGCTGGTGAAC |  |
| Adenovirus | Ad1 | + | TTCCCCATGGCICAYAACAC | 482 |
|  | Ad2 | - | CCCTGGTAKCCRATRTTGTA |  |
| Astrovirus | Mon269 | + | CAACTCAGGAAACAGGGTGT | 449 |
|  | Mon270 | - | TCAGATGCATTGTCATTGGT |  |
| Norovirus (genogroups I) | G1-SKF | + | CTGCCCGAATTYGTAAATGA | 330 |
|  | GI-SKR | - | CCAACCCARCCATTRTACA |  |
| Norovirus (genogroups II) | CoG2F | + | CARGARBCNATGTTYAGRTGGATGAG | 387 |
|  | G2-SKR | - | CCRCCNGCATRHCCRTTRTACAT |  |
| Sapovirus | SLV-5317 | + | CTCGCCACCTACRAWGCBTGGTT | 434 |
|  | SLV-5749 | - | CGGRCYTCAAAVSTACCBCCCCA |  |

Table S1-3. Primers and sequence information used in amplification for bacterial agents.

| **Pathogen** | **Primer** | **Sequence (5’-3’)** | **Amplicon (bp)** |
| --- | --- | --- | --- |
| EPEC | *eae*-F | TCAATGCAGTTCCGTTATCAGTT | 482 |
|  | *eae*-R | GTAAAGTCCGTTACCCCAACCTG |  |
|  | *bfp*-F | GGAAGTCAAATTCATGGGGGTAT | 300 |
|  | *bfp*-R | GGAATCAGACGCAGACTGGTAGT |  |
|  |  |  |  |
| ETEC | *lt*-F | GCACACGGAGCTCCTCAGTC | 218 |
|  | *lt*-R | TCCTTCATCCTTTCAATGGCTTT |  |
|  | *st*II-F | AAAGGAGAGCTTCGTCACATTTT | 129 |
|  | *st*II-R | AATGTCCGTCTTGCGTTAGGAC |  |
|  |  |  |  |
| EHEC | *eae*-F | TCAATGCAGTTCCGTTATCAGTT | 482 |
|  | *eae*-R | GTAAAGTCCGTTACCCCAACCTG |  |
|  | *stx*1-F | CAGTTAATGTGGTGGCGAAGG | 348 |
|  | *stx*1-R | CACCAGACAATGTAACCGCTG |  |
|  | *stx*2-F | ATCCTATTCCCGGGAGTTTACG | 584 |
|  | *stx*2-R | GCGTCATCGTATACACAGGAGC |  |
|  |  |  |  |
| EIEC | *virF*-F | AGCTCAGGCAATGAAACTTTGAC | 618 |
|  | *virF*-R | TGGGCTTGATATTCCGATAAGTC |  |
|  | *ipaH*-F | CTCGGCACGTTTTAATAGTCTGG | 933 |
|  | *ipaH*-R | GTGGAGAGCTGAAGTTTCTCTGC |  |
|  |  |  |  |
| EAEC | *aaf*II-F | CACAGGCAACTGAAATAAGTCTGG | 378 |
|  | *aaf*II-R | ATTCCCATGATGTCAAGCACTTC |  |
|  |  |  |  |
| *C. jejuni* | MapA-F | CTATTTTATTTTTGAGTGGTTGTG | 589 |
|  | MapA -R | GCTTTATTTGCCATTTGTTTTATTA |  |
|  |  |  |  |
| *C. coli* | CeuE-F | ATTTGAAAATTGCTCCAACTATG | 462 |
|  | CeuE-R | TGATTTTATTATTTGTAGCAGCG |  |
|  |  |  |  |
| *Y. enterocolitica* | *ail*-F | TAATGTGTACGCTGCGAG | 351 |
|  | *ail* -R | GACGTCTTACTTGCACTG |  |
|  | *ystA*-F | ATCGACACCAATAACCGCTGAG | 79 |
|  | *ystA*- R | CCAATCACTACTGACTTCGGCT |  |
|  | *ystB*-F | GTACATTAGGCCAAGAGACG | 146 |
|  | *ystB*-R | GCAACATACCTCACAACACC |  |
|  | *yadA*-F | CTTCAGATACTGGTGTCGCTGT | 849 |
|  | *yadA*-R | ATGCCTGACTAGAGCGATATCC | 759* |
|  | *virF*-F | GGCAGAACAGCAGTCAGACATA | 561 |
|  | *virF*- R | GGTGAGCATAGAGAATACGTCG |  |
|  |  |  |  |
| *Y. pseudotuberculosis* | *inv*-F | CGGTACGGCTCAAGTTAATCTG | 183 |
|  | *inv*-R | CCGTTCTCCAATGTACGTATCC |  |
|  | *yadA*-F | CTTCAGATACTGGTGTCGCTGT | 849 |
|  | *yadA*-R | ATGCCTGACTAGAGCGATATCC |  |
|  | *virF*-F | TCATGGCAGAACAGCAGTCAG | 590 |
|  | *virF*- R | ACTCATCTTACCATTAAGAAG |  |
|  | *ypmA*-F | CACTTTTCTCTGGAGTAGCG | 350 |
|  | *ypmA*-R | ACTCATCTTACCATTAAGAAG |  |
|  | *ypmB*-F | TTTCTGTCATTACTGACATTA | 453 |
|  | *ypmB*-R | CCTCTTTCCATCCATCTCTTA |  |
|  | *ypmC*-F | ACACTTTTCTCTGGAGTAGCG | 418 |
|  | *ypmC*-R | ACAGGACATTTCGTCA |  |

*The amplicon of Serotypes O:8 Y. enterocolitica.

**Table S2. The prevalence of 17 enteropathogens among febrile diarrheal patients by sex and age groups.**

|  | **Febrile** | **Afebrile** | **Sex** | | | | **Age** | | | | | | | | | |
| --- | --- | --- | --- | --- | --- | --- | --- | --- | --- | --- | --- | --- | --- | --- | --- | --- |
|  |  |  | **Male** | | **Female** | | **0-4 years** | | **5-17 years** | | **18-45 years** | | **46-59 years** | | **≥60 years** | |
|  |  |  | **Febrile** | **Afebrile** | **Febrile** | **Afebrile** | **Febrile** | **Afebrile** | **Febrile** | **Afebrile** | **Febrile** | **Afebrile** | **Febrile** | **Afebrile** | **Febrile** | **Afebrile** |
| **Any virus, n (%)** | 2163 (31.3) | 7875 (23.7)** | 1299 (31.6) | 4520 (24.8)** | 864 (30.8) | 3355 (22.4)** | 1699 (40.2) | 4094 (35.7)** | 96 (22.2) | 382 (20.2) | 204 (15.8) | 1732 (17.5) | 85 (18.0) | 831 (18.5) | 79 (15.9) | 836 (15.4) |
| Rotavirus A | 1086 (15.7) | 2630 (7.9)** | 655 (15.9) | 1512 (8.3)** | 431 (15.3) | 1118 (7.5)** | 996 (23.6) | 1861 (16.2)** | 29 (6.7) | 102 (5.4) | 29 (2.2) | 288 (2.9) | 14 (3.0) | 198 (4.4) | 18 (3.6) | 181 (3.3) |
| Norovirus | 792 (11.4) | 3723 (11.2) | 490 (11.9) | 2140 (11.8) | 302 (10.8) | 1583 (10.6) | 510 (12.1) | 1472 (12.8) | 44 (10.2) | 164 (8.7) | 135 (10.4) | 1088 (11.0) | 59 (12.5) | 503 (11.2) | 44 (8.9) | 496 (9.1) |
| Adenovirus | 275 (4.0) | 823 (2.5)** | 154 (3.7) | 485 (2.7)** | 121 (4.3) | 338 (2.3)** | 228 (5.4) | 615 (5.4) | 13 (3.0) | 48 (2.5) | 16 (1.2) | 71 (0.7) | 5 (1.1) | 38 (0.9) | 13 (2.6) | 51 (0.9)** |
| Astrovirus | 227 (3.3) | 803 (2.4)** | 133 (3.2) | 450 (2.5)** | 94 (3.4) | 353 (2.4)** | 173 (4.1) | 386 (3.4)* | 9 (2.1) | 68 (3.6) | 26 (2.0) | 202 (2.0) | 8 (1.7) | 66 (1.5) | 11 (2.2) | 81 (1.5) |
| Sapovirus | 90 (1.3) | 649 (2.0)** | 52 (1.3) | 386 (2.1)** | 38 (1.4) | 263 (1.8) | 61 (1.4) | 306 (2.7)** | 12 (2.8) | 37 (2.0) | 11 (0.9) | 176 (1.8)* | 4 (0.9) | 70 (1.6) | 2 (0.4) | 60 (1.1) |
| Rotavirus B | 15 (0.2) | 72 (0.2) | 10 (0.2) | 46 (0.3) | 5 (0.2) | 26 (0.2) | 13 (0.3) | 51 (0.4) | 0 (0.0) | 3 (0.1) | 1 (0.1) | 5 (0.1) | 0 (0.0) | 7 (0.1) | 1 (0.2) | 6 (0.1) |
| Rotavirus C | 13 (0.2) | 52 (0.2) | 11 (0.3) | 35 (0.2) | 2 (0.2) | 17 (0.1) | 9 (0.2) | 24 (0.2) | 1 (0.2) | 3 (0.2) | 2 (0.2) | 10 (0.1) | 1 (0.2) | 5 (0.1) | 0 (0.0) | 10 (0.2) |
| **Any bacteria, n (%)** | 1401 (20.2) | 4612 (13.9)** | 829 (20.2) | 2380 (13.1)** | 572 (20.4) | 2232 (14.9)** | 607 (14.4) | 1080 (9.4)** | 83 (19.2) | 267 (14.1)** | 439 (33.9) | 1852 (18.7)** | 142 (30.1) | 669 (14.9)** | 130 (26.2) | 744 (13.7)** |
| NTS | 611 (8.8) | 1042 (3.1)** | 348 (8.5) | 549 (3.0)** | 263 (9.4) | 493 (3.3)** | 361 (8.5) | 369 (3.2)** | 29 (6.7) | 50 (2.7)** | 117 (9.0) | 311 (3.1)** | 53 (11.2) | 147 (3.3)** | 51 (10.3) | 165 (3.0)** |
| DEC | 524 (7.6) | 2321 (7.0) | 313 (7.6) | 1195 (6.6)* | 211 (7.5) | 1126 (7.5) | 189 (4.5) | 579 (5.0) | 33 (7.6) | 121 (6.4) | 191 (14.8) | 951 (9.6)** | 53 (11.2) | 312 (7.0)** | 58 (11.7) | 358 (6.6)** |
| *V. parahaemolyticus* | 97 (1.4) | 582 (1.8)* | 56 (1.4) | 276 (1.5) | 41 (1.5) | 306 (2.0)* | 1 (0.0) | 4 (0.0) | 0 (0.0) | 17 (0.1) | 61 (4.7) | 367 (3.7) | 21 (4.5) | 115 (2.6)* | 14 (2.8) | 79 (1.5)* |
| *Shigella* | 96 (1.4) | 158 (0.5)** | 67 (1.6) | 81 (0.4)** | 29 (1.0) | 77 (0.5)** | 44 (1.0) | 34 (0.3)** | 11 (2.6) | 28 (1.5) | 37 (2.9) | 56 (0.6)** | 4 (0.9) | 15 (0.3) | 0 (0.0) | 25 (0.5) |
| *C. jejuni* | 91 (1.3) | 244 (0.7)** | 56 (1.4) | 145 (0.8)** | 35 (1.3) | 99 (0.7)** | 10 (0.2) | 73 (0.6)** | 12 (2.8) | 24 (1.3)* | 49 (3.8) | 79 (0.8)** | 13 (2.8) | 28 (0.6)** | 7 (1.4) | 40 (0.7) |
| *P. shigelloides* | 26 (0.4) | 144 (0.4) | 16 (0.4) | 77 (0.4) | 10 (0.4) | 67 (0.5) | 1 (0.0) | 2 (0.0) | 0 (0.0) | 7 (0.4) | 20 (1.6) | 76 (0.8)** | 3 (0.6) | 29 (0.7) | 2 (0.4) | 30 (0.6) |
| *A. hydrophila* | 19 (0.3) | 268 (0.8)** | 11 (0.3) | 136 (0.8)** | 8 (0.3) | 132 (0.9)** | 6 (0.1) | 30 (0.3) | 2 (0.5) | 15 (0.8) | 8 (0.6) | 112 (1.1) | 2 (0.4) | 41 (0.9) | 1 (0.2) | 70 (1.3) |
| *C. coli* | 16 (0.2) | 44 (0.1) | 9 (0.2) | 21 (0.1) | 7 (0.3) | 23 (0.2) | 4 (0.1) | 20 (0.2) | 0 (0.0) | 3 (0.2) | 9 (0.7) | 10 (0.1)** | 1 (0.2) | 6 (0.1) | 2 (0.4) | 5 (0.1) |
| *Y. enterocolitica* | 14 (0.2) | 51 (0.2) | 9 (0.2) | 27 (0.2) | 5 (0.2) | 24 (0.2) | 7 (0.2) | 20 (0.2) | 1 (0.2) | 9 (0.5) | 4 (0.3) | 10 (0.1) | 1 (0.2) | 5 (0.1) | 1 (0.2) | 7 (0.1) |
| *V. cholerae* | 2 (0.0) | 42 (0.1)* | 1 (0.0) | 18 (0.1) | 1 (0.0) | 24 (0.2) | 0 (0.0) | 0 (0.00) | 0 (0.0) | 1 (0.1) | 0 (0.0) | 30 (0.3)* | 1 (0.2) | 3 (0.1) | 1 (0.2) | 8 (0.2) |
| Co-infection |  |  |  |  |  |  |  |  |  |  |  |  |  |  |  |  |
| Co-infection with ≥ 2 viruses | 290 (4.2) | 789 (2.4)** | 180 (4.4) | 473 (2.6)** | 110 (3.9) | 316 (2.1)** | 250 (5.9) | 550 (4.8)** | 11 (2.6) | 38 (2.0) | 14 (1.1) | 101 (1.0) | 6 (1.3) | 54 (1.2) | 9 (1.8) | 46 (0.9)* |
| Co-infection with ≥ 2 bacteria | 90 (1.3) | 272 (0.8)** | 54 (1.3) | 139 (0.8)** | 36 (1.3) | 133 (0.9) | 16 (0.4) | 48 (0.4) | 5 (1.2) | 8 (0.4) | 52 (4.0) | 144 (1.5)** | 10 (2.1) | 31 (0.7)** | 7 (1.4) | 41 (0.8) |
| Viral-bacterial co-infection | 220 (3.2) | 758 (2.3)** | 138 (3.4) | 402 (2.2)** | 82 (2.9) | 356 (2.4) | 152 (3.6) | 281 (2.5)** | 13 (3.0) | 36 (1.9) | 33 (2.6) | 244 (2.5) | 12 (2.5) | 109 (2.4) | 10 (2.0) | 88 (1.6) |

Abbreviations: DEC, diarrheagenic Escherichia coli; NTS, nontyphoidal Salmonella.

Chi square test or Fisher exact test for comparisons of detection between febrile and afebrile cases. The significant difference (chi-square test or Fisher’s exact test were two-sided) found within the group was showed by asterisk (* *p*<0.05; ** *p*<0.01). Red texts indicate that febrile cases were of significantly higher prevalence than afebrile patients. Blue texts indicate the significantly lower prevalence of febrile patients.

**Table S3. The ratio of prevalence (febrile patients vs afebrile patients) for each pathogen by sex and age groups.**

|  | **All patients** | **Age groups, years** | | | | |
| --- | --- | --- | --- | --- | --- | --- |
|  |  | **0–4 years** | **5–17 years** | **18–45 years** | **46–59 years** | **≥ 60 years** |
| **Any virus positive detection** | 1.32 | 1.13 | 1.10 | 0.90 | 0.97 | 1.03 |
| Rotavirus A | 1.98 | 1.45 | 1.24 | 0.77 | 0.67 | 1.09 |
| Norovirus | 1.02 | 0.94 | 1.17 | 0.95 | 1.12 | 0.97 |
| Adenovirus | 1.60 | 1.01 | 1.19 | 1.73 | 1.25 | 2.78 |
| Astrovirus | 1.36 | 1.22 | 0.58 | 0.99 | 1.15 | 1.48 |
| Sapovirus | 0.67 | 0.54 | 1.42 | 0.48 | 0.54 | 0.36 |
| Rotavirus B | 1.00 | 0.69 | 0 | 1.53 | 0 | 1.82 |
| Rotavirus C | 1.20 | 1.02 | 1.46 | 1.53 | 1.90 | 0 |
| **Any bacteria positive detection** | 1.46 | 1.53 | 1.36 | 1.82 | 2.02 | 1.91 |
| NTS | 2.81 | 2.66 | 2.54 | 2.89 | 3.43 | 3.37 |
| DEC | 1.08 | 0.89 | 1.19 | 1.54 | 1.62 | 1.77 |
| *V. parahaemolyticus* | 0.80 | 0.68 | 0 | 1.28 | 1.74 | 1.93 |
| *Shigella* | 2.91 | 3.51 | 1.72 | 5.07 | 2.54 | 0 |
| *C. jejuni* | 1.79 | 0.37 | 2.19 | 4.76 | 4.41 | 1.91 |
| *P. shigelloides* | 0.87 | 1.36 | 0 | 2.02 | 0.98 | 0.73 |
| *A. hydrophila* | 0.34 | 0.54 | 0.58 | 0.55 | 0.46 | 0.16 |
| *C. coli* | 1.74 | 0.54 | 0 | 6.90 | 1.58 | 4.37 |
| *Y. enterocolitica* | 1.32 | 0.95 | 0.49 | 3.07 | 1.90 | 1.56 |
| *V. cholerae* | 0.23 | - | 0 | 0 | 3.17 | 1.36 |
| **Co-infection positive detection** |  |  |  |  |  |  |
| Co-infection with ≥ 2 viruses | 1.76 | 1.23 | 1.27 | 1.06 | 1.06 | 2.14 |
| Co-infection with ≥ 2 bacteria | 1.59 | 1.27 | 2.73 | 2.77 | 3.07 | 1.86 |
| Viral-bacterial co-infection | 1.60 | 1.30 | 1.48 | 1.60 | 1.41 | 1.71 |

Abbreviations: DEC, diarrheagenic Escherichia coli; NTS, nontyphoidal Salmonella.

The ratio of prevalence (RP) was calculated as prevalence of febrile patients / prevalence of afebrile patients.

**Table S4. The prevalence of co-infection between two enteropathogens among 17 enteropathogens.**

|  | **Rotavirus A** | **Norovirus** | **Adenovirus** | **Astrovirus** | **Sapovirus** | **Rotavirus B** | **Rotavirus C** | **NTS** | **DEC** | ***V. parahaemolyticus*** | ***Shigella*** | ***C. jejuni*** | ***P. shigelloides*** | ***A. hydrophila*** | ***C. coli*** | ***Y. enterocolitica*** |
| --- | --- | --- | --- | --- | --- | --- | --- | --- | --- | --- | --- | --- | --- | --- | --- | --- |
| Norovirus | 260(1.8) / 497(0.9)** |  |  |  |  |  |  |  |  |  |  |  |  |  |  |  |
| Adenovirus | 126(0.9) / 276(0.5)** | 152(0.9) / 343(0.5)** |  |  |  |  |  |  |  |  |  |  |  |  |  |  |
| Astrovirus | 101(0.7) / 173(0.3)** | 119(0.7) / 288(0.4)** | 84(0.5) / 140(0.2)** |  |  |  |  |  |  |  |  |  |  |  |  |  |
| Sapovirus | 44(0.3) / 95(0.2)** | 91(0.6) / 174(0.3)** | 76(0.5) / 112(0.2)** | 67(0.4) / 93(0.1)** |  |  |  |  |  |  |  |  |  |  |  |  |
| Rotavirus B | 37(0.3) / 47(0.1)** | 2 / 13 | 2 / 4 | 2 / 6 | 0 / 4 |  |  |  |  |  |  |  |  |  |  |  |
| Rotavirus C | 32(0.3) / 18** | 5 / 6* | 3 / 4 | 7(0.1) / 4** | 1 / 2 | 56(0.4) / 28** |  |  |  |  |  |  |  |  |  |  |
| NTS | 51(0.6) / 40(0.1)** | 68(0.6) / 137(0.3)** | 29(0.3) / 20** | 9(0.1) / 17* | 6(0.1) / 17 | 0 / 1 | 3 / 0** |  |  |  |  |  |  |  |  |  |
| DEC | 36(0.4) / 102(0.3) | 83(0.8) / 431(0.9) | 21(0.2) / 80(0.2) | 13(0.1) / 72(0.2) | 11(0.1) / 83(0.2) | 0 / 1 | 0 / 5 | 79(0.4) / 151(0.2)** |  |  |  |  |  |  |  |  |
| *V. parahaemolyticus* | 0 / 6 | 5(0.1) / 44(0.1) | 1 / 10 | 2 / 12 | 0 / 9 | 0 / 0 | 0 / 0 | 3 / 52(0.1)* | 20(0.1) / 90(0.1) |  |  |  |  |  |  |  |
| *Shigella* | 5(0.1) / 3** | 11(0.1) / 10** | 6(0.1) / 9* | 1 / 2(0) | 1 / 4 | 0 / 0 | 0 / 0 | 6 / 14 | 29(0.2) / 39(0.1)** | 0 / 7 |  |  |  |  |  |  |
| *C. jejuni* | 4(0.1) / 5 | 11(0.1) / 53(0.1) | 4 / 6 | 1 / 9 | 1 / 11 | 0 / 0 | 0 / 0 | 4 / 22 | 26(0.2) / 38(0.1)** | 2 / 7 | 0 / 2 |  |  |  |  |  |
| *P. shigelloides* | 1 / 2 | 7(0.1) / 34(0.1) | 0 / 2 | 0 / 0 | 1 / 2 | 0 / 1 | 0 / 0 | 8(0.1) / 22 | 7 / 47(0.1) | 4 / 31 | 0 / 2 | 1 / 5 |  |  |  |  |
| *A. hydrophila* | 1 / 12 | 3 / 39(0.1) | 2 / 2 | 4 / 3* | 0 / 1 | 0 / 0 | 0 / 0 | 8(0.1) / 28 | 9(0.1) / 61(0.1) | 1 / 22 | 5 / 11 | 3 / 6 | 1 / 14 |  |  |  |
| *C. coli* | 2 / 2 | 4 / 6 | 0 / 1 | 0 / 4 | 1 / 1 | 0 / 0 | 0 / 0 | 3 / 4 | 6 / 11 | 0 / 3 | 0 / 1 | 1 / 8 | 0 / 2 | 0 / 2 |  |  |
| *Y. enterocolitica* | 0 / 2 | 2 / 9 | 0 / 2 | 0 / 4 | 1 / 0 | 0 / 0 | 0 / 0 | 0 / 4 | 5 / 14 | 1 / 4 | 0 / 2 | 0 / 1 | 0 / 2 | 0 / 1 | 1 / 1 |  |
| *V. cholerae* | 0 / 2 | 0 / 2 | 0 / 0 | 0 / 0 | 0 / 0 | 0 / 0 | 0 / 0 | 1 / 9 | 0 / 13 | 0 / 5 | 0 / 3 | 0 / 0 | 0 / 5 | 0 / 1 | 0 / 0 | 0 / 0 |

Abbreviations: DEC, diarrheagenic Escherichia coli; NTS, nontyphoidal Salmonella.

Chi square test or Fisher exact test for comparisons of detection between febrile and afebrile cases. The significant difference (chi-square test or Fisher’s exact test were two-sided) found within the group was showed by asterisk (* *p*<0.05; ** *p*<0.01). Red texts indicate that febrile cases were of significantly higher prevalence than afebrile patients. Blue texts indicate the significantly lower prevalence of febrile patients.

**Table S5. Factors associated with fever occurrence among diarrheal patients by logistic regression analysis.**

|  | **Children** | | |  | **Adults** | | |
| --- | --- | --- | --- | --- | --- | --- | --- |
|  | **Number** | **Adjusted *OR* (95% *CI*)** | ***P*** |  | **Number** | **Adjusted *OR* (95% *CI*)** | ***P*** |
| Sex |  |  |  |  |  |  |  |
| Female | 6080 |  |  |  | 10646 | 1 | - |
| Male | 9662 |  |  |  | 11066 | 1.26 (1.15, 1.38) | < 0.001 |
| Age* (5 years) | 15742 | 0.84 (0.80, 0.89) | < 0.001 |  | 21712 | 0.96 (0.94, 0.97) | < 0.001 |
| Season |  |  |  |  |  |  |  |
| Spring | 4659 |  |  |  | 7145 | 1 | - |
| Summer | 4252 |  |  |  | 7297 | 2.07 (1.77, 2.43) | < 0.001 |
| Autumn | 2701 |  |  |  | 3820 | 2.04 (1.75, 2.40) | < 0.001 |
| Winter | 4130 |  |  |  | 3450 | 1.43 (1.18, 1.72) | < 0.001 |
| Residence |  |  |  |  |  |  |  |
| Urban | 11898 | 1 | - |  |  |  |  |
| Rural | 3844 | 1.36 (1.26, 1.48) | < 0.001 |  | 2571 |  |  |
| Virus ^a^ |  |  |  |  |  |  |  |
| Rotavirus A | 2730 | 1.60 (1.46, 1.75) | < 0.001 |  | 719 | 0.91 (0.68, 1.21) | 0.549 |
| Norovirus | 1960 | 0.95 (0.85, 1.07) | 0.402 |  | 2311 |  |  |
| Adenovirus | 723 | 0.94 (0.77, 1.14) | 0.521 |  | 192 | 1.61 (1.02, 2.42) | 0.030 |
| Astrovirus | 566 |  |  |  | 385 | 1.48 (1.06, 2.03) | 0.018 |
| Sapovirus | 337 | 0.67 (0.51, 0.88) | 0.005 |  | 320 | 0.53 (0.31, 0.84) | 0.011 |
| Bacteria ^a^ |  |  |  |  |  |  |  |
| NTS | 709 | 2.95 (2.52, 3.45) | < 0.001 |  | 833 | 3.59 (3.03, 4.23) | < 0.001 |
| DEC | 738 | 1.03 (0.86, 1.21) | 0.776 |  | 1915 | 1.64 (1.43, 1.87) | < 0.001 |
| *V. parahaemolyticus* | 21 |  |  |  | 649 | 1.45 (1.15, 1.82) | 0.001 |
| *Shigella* | 105 | 2.93 (1.98, 4.33) | <0.001 |  | 129 | 3.34 (2.24, 4.89) | < 0.001 |
| *C. jejuni* | 118 | 0.62 (0.36, 1.02) | 0.073 |  | 126 | 4.40 (3.23, 5.92) | < 0.001 |
| *P. shigelloides* | 10 |  |  |  | 159 |  |  |
| *A. hydrophila* | 48 |  |  |  | 233 | 0.35 (0.17, 0.63) | 0.001 |

Abbreviations: *OR*=odds ratio; 95% *CI*=95% confidence interval; DEC=diarrheagenic *Escherichia coli*; NTS=nontyphoidal *Salmonella*.

Z test for comparisons among patients for different groups. All statistical tests were two-sided and *P* < 0.05 was statistically significant. ^a^ The numbers of patients with specific pathogen was shown. ^b^ Due to excessive co-infections (66 co-infections), all of co-infections cannot be listed in table and only them of significance in multivariate analysis were listed.

* The *OR* value represented by each year of age reduction is the reciprocal of the *OR* value of the age in the table.
